# Modelling the impacts of climate change on agrochemical fate and transport by water on a catchment scale

**DOI:** 10.1016/j.heliyon.2024.e35669

**Published:** 2024-08-03

**Authors:** Poornima Nagesh, Matthias Gassmann, Josef Eitzinger, Hugo J. de Boer, Oreane Y. Edelenbosch, Detlef P. van Vuuren, Stefan C. Dekker

**Affiliations:** a Copernicus Institute of Sustainable Development, Utrecht University, Utrecht, the Netherlands; b Department of Hydrology and Substance Balance, University of Kassel, Kassel, Germany; c Institute of Meteorology and Climatology, University of Natural Resources and Life Sciences, Vienna, BOKU, Austria; d PBL Netherlands Environmental Assessment Agency, the Netherlands

## Abstract

The export of agrochemicals and their transformation products (TPs) following their application in the agricultural fields poses a threat to water quality. Future changes in climatic conditions (e.g. extreme weather events such as heavy rainfall or extended dry periods) could alter the degradation and mobility of agrochemicals. In this research, we use an integrated modelling framework to understand the impact of extreme climate events on the fate and transport of the agrochemical S-Metolachlor and two of its TPs (M-OXA, Metolachlor Oxanilic Acid and M-ESA, Metolachlor Ethyl Sulfonic Acid). This is done by coupling climate model outputs to the Zin-AgriTra agrochemical reactive transport model in four simulation scenarios. 1) Reference (2015–2018), 2) Very dry (2038–2041), 3) Very wet (2054–2057) and 4) High temperature (2096–2099) conditions of a selected RCP8.5 based regional climate scenario. The modelling framework is tested on an agricultural catchment, Wulka, in Burgenland, Austria. The model results indicate that 13–14 % of applied S-Metolachlor is retained in the soil, and around 85 % is degraded into TPs in the different scenarios. In very dry and high-temperature scenarios, degradation is higher, and hence, there is less S-Metolachlor in the soil. However, a large share of formed M-OXA and M-ESA are retained in the soil, which is transported via overland and groundwater flow, leading to a build-up effect in M-OXA and M-ESA river concentrations over the years. Though a small share of S-Metolachlor and TPs are transported to rivers, their river export is affected by the intensity and amount of rainfall. The very wet and high-temperature scenarios show higher S-Metolachlor and TP concentrations at the catchment outlet due to higher river discharge. The reference scenario shows higher river peak concentrations associated with higher overland flow caused by measured hourly rainfall compared to disaggregated daily precipitation data in the other scenarios.

## Highlights

• Fate and transport of agrochemicals and TPs under extreme climate events is studied.
• Climate model outputs are coupled to Zin-AgriTra model in four simulation scenarios.
• Very wet scenario shows higher S-metolachlor river concentrations.
• High-temperature scenarios show higher agrochemical degradation.
• Intense hourly rainfall causes peaks in agrochemical river concentrations.

## 1 Introduction

The use of agrochemicals helps control various pests and diseases that threaten crops and improve food quality. However, agrochemicals dissipate with time and degrade into Transformation Products (TPs) [1,2]. In recent years, there has been growing attention concerning TPs as they can be more persistent, mobile, and toxic than the parent agrochemical [3,4]. They are often detected in concentrations higher than their parent agrochemical and with higher detection frequency [3,5,6]. The transport of agrochemicals and TPs after application from the agricultural fields threatens the ground and surface water quality in many regions of the world [5,7].

Once applied, a large share of agrochemicals and their TPs are retained in the soil, infiltrated into groundwater, or transported to surface waters. The concentrations of agrochemicals in the different environmental compartments depend on numerous factors, including their chemical properties and local environmental conditions. Intense precipitation events are shown to impact agrochemical fate and transport substantially [[8], [9], [10]]. Climate factors also play a role: elevated temperatures can lead to higher degradation rates of agrochemicals [11], while associated droughts can increase the persistence of agrochemicals due to suppressed degradation [12]. Global climate change is associated with significant changes in factors like long-term weather characteristics and short-term extremes [13], which can further affect agrochemicals. Changes in climatic conditions pose a direct effect on both the quality and quantity of water resources. Increasing occurrence of extreme weather events, such as floods and droughts, will likely alter the mobility of agrochemicals [13]. Direct effects include increased agrochemical leaching and overland and subsurface flows due to increased and frequent rainfall [11,14,15]. Alterations in soil characteristics (organic carbon or moisture content) and hydrology can change agrochemical sorption, transport, and dilution potentials in the surface water [13]. Climate change can indirectly influence agrochemicals by altering agricultural land use for intensive agricultural production [16], which varies diffuse emissions to rivers [17]. Adaptation to climatic conditions alters the timing of crop cultivation, the crop type grown, and new and higher survival rates in weeds and pests [[18], [19], [20]]. The use of agrochemicals can vary with rising pest pressure and growing demand for agricultural products [21]. With changing agricultural land use alone, agrochemical use and emissions are projected to increase by 2050 [22].

It is essential to understand how climate change and induced extreme events affect agrochemicals and TPs fate and transport processes in the future. For the assessment of agrochemicals and their impacts on water resources and climate extremes, a variety of modelling approaches are applied, including SWAT [[23], [24], [25], [26]], GIS modelling [27], MACRO [11], DynAplus [9], dynamic (IV) multimedia fate model [8] and life cycle assessment methods [28]. However, most modelling approaches concentrate on the total amount or concentration of agrochemicals exported from a catchment and often neglect the transformation products [2]. Additionally, they often focus on daily, monthly or cropping season dynamics and do not consider off-season and post-yearly effects of extreme climate events.

Since an increasing number of agrochemicals and TPs have been detected in various environmental compartments, a more comprehensive understanding of their fate and transport in future extreme climate events is needed. However, such an approach has not yet been applied to evaluate agrochemical emissions in the context of analysing the impacts of extreme climate events. In order to understand the future risk posed by agrochemical fate and transport to the environment, it is essential to integrate an agrochemical fate and transport model with climate change drivers (e.g., temperature, precipitation) that influence their fate and transport. For this research, we used a well-used process-based hydrological model, ZIN-AgriTra, that can simulate the fate and transport of agrochemicals and their TPs from the catchment.

Our current research aims to understand agrochemical dynamics and their TPs under climate change. We use an integrated modelling framework to include climatic extremes in four simulation scenarios up to the year 2100 to quantify agrochemical fate and transport to different environmental compartments. We used ZIN-AgriTra, a fully distributed agrochemical fate and transport model on a catchment scale, to simulate agrochemicals and TP export to environmental compartments at daily time steps [2,30]. The integrated modelling framework links the output of Representative Concentration Pathways (RCP) presenting climate extremes to the agrochemical fate model (Zin-AgriTra). As the fate and transport of agrochemicals and TPs are highly dependent on local environmental conditions, the framework is tested on an agricultural catchment in Burgenland, Austria, with the well-researched herbicide S-Metolachlor.

## 2 Methods

### 2.1 Overview of the modelling framework

A coupled, integrated modelling framework is used to understand the influence of climate change on agrochemical fate and transport by water from crop canopies and soil surface through the soil and belowground flows and overland flow to surface waters. The integrated modelling framework couples the climate model results of the RACMO22E Regional Climate Model (RCM) [31] to the ZIN-AgriTra model as boundary conditions [2,30]. RACMO22E is an RCM developed by the Royal Netherlands Meteorological Institute (KNMI) that generates climate projections [31] using Representative concentration pathways (RCPs) with a spatial resolution of 12 km to develop Austrian climate scenarios (ÖKS15). The ÖKS15 dataset provides projections made using the RCPs and the EURO‐CORDEX RCM ensemble [32,33] developed by combining six RCMs with five global climate models to provide a total of thirteen different climate projections with a horizontal resolution of 12.5 km for two RCPs, RCP 4.5 and RCP 8.5, covering the 1971–2100 period. RACMO22E is one of the six RCMs generating climate projections for RCP4.5 and 8.5. Further, a high resolution of 1 km grid size in the ÖKS15 was achieved by interpolating the RCM projections onto the GPARD1 grid of observational data (1 km grid size), including bias corrections.

#### 2.1.1 Zin-AgriTra model

**Fig. 1:**
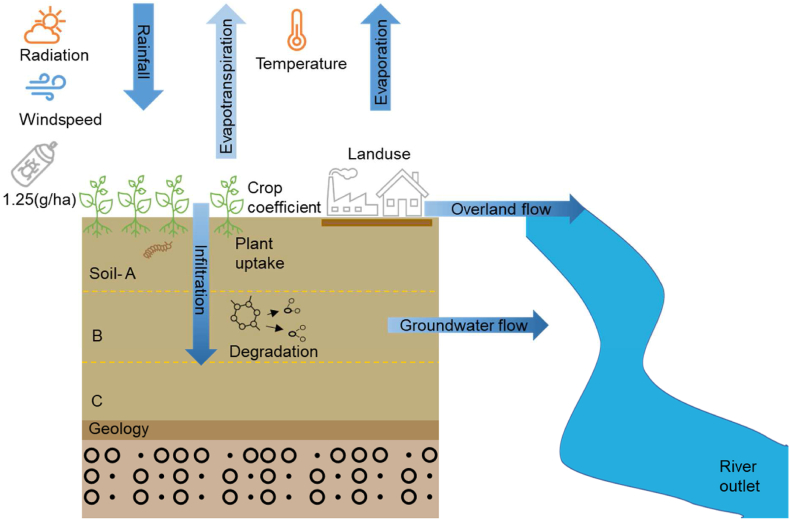
Represents the agrochemical use, fate, and transport model scheme in the Zin-AgriTra model for different environmental compartments with input parameters (based on Gaβmann [30], Gassmann et al. [2,7] and Gassmann [34]).

The ZIN-AgriTra is a catchment scale reactive transport model and can simulate agrochemical and TP export from agricultural catchments [2,7,30]. The model calculates agrochemical and TPs exported to different environmental compartments, accounting for catchment hydrological processes, erosion and sediment transport, agrochemical sorption, and agrochemical fate processes and transport under specified boundary conditions (Fig. 1). The hydrological fluxes modelled include evapotranspiration, groundwater flow (soil matrix flow and preferential flow in soil macropores), overland flow and channel routing [30]. Erosion processes constitute sheet and rill erosion and transport capacity [30]. The sorption processes describe the agrochemical attachment to soil particles and are calculated with either linear (eq. (1)) or Freundlich isotherm, explaining the equilibrium partitioning between dissolved and adsorbed pollutants. The sorption kinetics is calculated with a spontaneous adsorption equilibrium.

(1) $$C_{e\left(sorbed\right)}=K_{d}*C_{e\left(solved\right)}$$

in which:

K_d_ – Partitioning coefficient (l/g)

K_f_ – Freundlich sorption coefficient (mg/g)

C_e(sorbed)_ – equilibrium adsorbed concentration of agrochemical (mg/g)

C_e(solved)_ – equilibrium dissolved concentration of agrochemical (mg/l)

Agrochemicals are either applied on the soil or the plant surface. For plant-applied agrochemicals, the wash-off process is important, especially the amount after the first rainfall event. Agrochemicals applied at the plant surface are washed off to a certain fraction, f_wash-off_. A fraction of the applied agrochemical is deposited at the soil surface, which is specified by the plant-application fraction f_plant−soil_. The degradation and wash-off of applied agrochemicals and TPs are calculated as below. The degradation in the soil is influenced by environmental factors such as soil moisture, soil temperature, soil depth, pH, clay content and organic matter content. The influence of soil temperature on degradation is calculated based on the Arrhenius equation [34]. Degradation of the mass of the parent compound (PC) is calculated by the mass over time applied in the field minus the degradation and minus the loss by runoff and infiltration to deeper soil:

(3) $$\frac{\partial m_{PC}}{\partial t}=m_{app}-\left(\frac{\ln\left(2\right)}{{DT50}_{PC}}m_{PC}\right)-m_{PC,runoff}-m_{PC,\mathrm{Inf}}$$

Degradation of the transformation product (TP) is calculated equally to that of the PC. The only input is the formation fraction of TP from the PC.

(4) $$\frac{\partial m_{TP}}{\partial t}={ff}_{PC-TP}\left(\frac{\ln\left(2\right)}{{DT50}_{PC}}m_{PC}\right)-\left(\frac{\ln\left(2\right)}{{DT50}_{TP}}m_{TP}\right)-m_{TPrunoff}-m_{TP,\mathrm{Inf}}$$

with:

m_app_ – agrochemical mass applied in the field (g/ha)

m_(PC, Runoff)_ & m_(TP, Runoff)_ mass exported towards the river (g/ha)

m_(PC, Inf)_ & m_(TP, Runoff)_ – mass infiltrating into deeper soil (g/ha)

PC – Parent Compound.

TP – Transformation Product

ff_PC-TP_ – formation fraction of TP from PC.

*DT*50_PC_ – transformation half-lives of PC (days)

*DT*50 _*TP*_ – transformation half-lives of TP (days)

The transport of the agrochemicals is calculated spatial explicitly as mass transport from cell to cell. Here, it is assumed that the fraction of dissolved agrochemicals being transported is equal to the fraction of transported water. Agrochemicals can be transported attached to suspended sediment in overland flow and in the river. Agrochemicals can further enter the soil matrix and soil macropores in dissolved form by infiltration. Both agrochemicals and TPs can be exported to the river via macropore flow, matrix flow and surface runoff [30].

### 2.2 Catchment characteristics

**Fig. 2:**
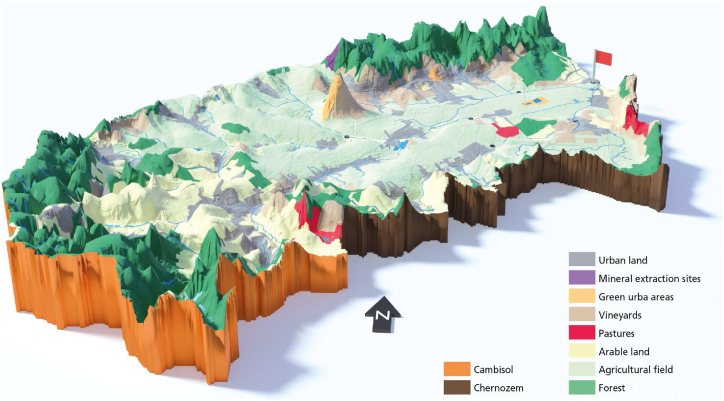
The Wulka River catchment with the land use and modelled catchment outlet. The length of the catchment is approximately 36 km.

The modelling framework was applied to a case study area, the Wulka catchment in Burgenland, Austria. The Wulka River is in the eastern part of Austria, discharging into the lake Neusiedler see. The Wulka River is along areas cultivating maize and wheat and constitutes a potential source of pollution to the lake. The total area of the catchment is 397 km^2^, with 54 % of the cropland area (Table 1). The Wulka catchment, with its land use and modelled outflow points, is presented in Fig. 2.

**Table 1:** Properties of the agricultural catchment.

Attribute		Unit	Wulka	Reference
Area		km^2^	397	
Mean slope		%	8	[35,36]
Dominant soil type		–	Silty loam soil	[35,36]
Land use	**Arable land**	%	54	[35,36]
	**Pastures**	%	12	[35,36]
	**Forests**	%	28	[35,36]
	**Urban areas**	%	6	[35,36]
Average precipitation		mm/a	625	[35,36]
Average river discharge		m^3^/s	1.23	[36]

### 2.3 Model calibration

#### 2.3.1 Climate scenarios and reference period

The simulation scenarios applied in the modelling framework are based on downscaled climate model run results for the RCP 8.5 emission scenario (climate scenario) of the 21st century. As the current research focuses on understanding the agrochemical and TPs fate and transport under extreme climatic conditions, we specifically use RCP 8.5, which represents a higher variance up to 2100 in predicted precipitation and temperatures than RCP 4.5. The extreme climatic conditions from 2020 to 2100 were analysed based on cumulative monthly precipitation and average temperature during the growing season to select three extreme scenarios (See Supplemental material-B.1 Climate statistics). The four simulation scenarios represent 1) Reference (S1 and S1_eq), 2) Very dry (S2), 3) Very wet (S3) and 4) High temperature (S4) condition periods of three consecutive years out of the climate model run and the "Reference” observed period represented by the meteorological station data for 2015–2018 (Table 2). An additional simulation scenario run was conducted for the reference scenario, S1_eq, using daily disaggregated rainfall to get a realistic comparison with the climate simulation scenarios. The very dry scenario presents consecutive dry years from 2038 to 2041 with below-average rainfall in that decade. The very wet scenario from 2054 to 2057 represents years with above-average and high-frequency rainfall in the decade. The extreme temperature scenario from 2096 to 2099 shows above-average monthly and yearly temperatures in the decade. This scenario also represents years with a higher frequency of above-average rainfall. To get realistic initial conditions for hydrological modelling, we performed a 2-year warm-up model run using twice the observed data from 1 January to December 31, 2015 of the meteorological station Eisenstadt [37]. The 2-year warm-up model run is used as a baseline run for the four scenarios to represent the same initial state. The current agricultural land use, soil, geology, and agrochemical application guidelines are assumed to be the same in all four climate scenarios.

**Table 2:** Overview of the simulation scenarios.

Simulation scenarios	Years	Average daily rainfall per year (mm)	Number of days with rainfall	Number of days with >30 mm rainfall	Average yearly temperature	Number of days with high mean temperature (>25^0^C)
**S1 (reference,** observed period**)**	2015	1.48	107	2	12.47	27
	2016	1.99	136	2	11.99	7
	2017	1.69	154	2	12.04	22
**S2 (very dry,** scenario period**)**	2038	1.09	130	0	13.22	12
	2039	1.26	149	1	13.88	40
	2040	1.31	146	0	11.68	17
**S3 (very wet,** scenario period**)**	2054	2.16	165	2	12.46	8
	2055	1.57	147	2	11.04	8
	2056	2.09	151	4	12.54	18
**S4 (high temperature,** scenario period**)**	2096	1.73	147	0	15.27	48
	2097	2.08	150	3	14.82	44
	2098	1.69	137	2	15.67	53

The climate data for the four climate scenarios are used as input to the Zin-AgriTra model. The model simulates hydrological and agrochemical fate and transport processes based on precipitation, radiation, average temperature, humidity, wind speed and evapotranspiration input on hourly or daily time scales. The meteorological data on daily and hourly time steps for 2015–2018 were obtained from the meteorological station at Eisenstadt [37]. The climate scenario data on daily time steps for 2038–2041, 2054–2057 and 2096–2099 were obtained from the RACMO22E model [31] results at daily resolution. Given the catchment scale, we did not downscale the climate scenario results spatially. For temporal downscaling, the daily precipitation and radiation were disaggregated into hourly time steps. Precipitation disaggregation was conducted using an equal distribution method. Disaggregated radiation data was obtained from the research project SECURES [38,39]. Evapotranspiration was calculated using the Penman-Monteith calculator using the FAO (Food and Agriculture Organization) method [[40], [41], [42]]. Relative humidity for future scenarios was obtained by calculating the monthly average of historic relative humidity data from 2000 to 2022.

#### 2.3.2 Spatial data

The land use of the Wulka catchment was extracted from the Corine 2018 land cover database [43]. Catchment geological data was extracted from the Austrian government's open data platform [44]. Soil type and depth files were created from the European soil data centre [[45], [46], [47]]. Monthly crop coefficient values for maize were obtained from Eitzinger et al. [48] and Nistor et al. [49]. The crop calendar for maize in Austria was derived from the FAO crop calendar [50]. The DEM data were used to delineate the watershed, calculate the stream layer, and determine the pour points. All the spatial data were further processed to the Wulka catchment and gridded to 200*200-m grid size. The geology, land use and soil property files were created to support this spatial data (See Supplemental material -B.2 Geology properties, B.3 Land use properties and B.5 Soil properties).

#### 2.3.3 Agrochemical application

The herbicide S-Metolachlor with the transformation products Metolachlor Oxanilic Acid (M-OXA) and Metolachlor Ethyl Sulfonic Acid (M-ESA) was used to model the agrochemical fate and transport. S-metolachlor (2-chloro-N-(2-ethyl-6-methylphenyl)-N-[(1S)-2-methoxy-1-methylethyl]acet-amide) is a selective chloroacetanilide herbicide and is one of the three most commonly used herbicides worldwide [51]. S-metolachlor is commonly used for a wide range of crops, including maize, sorghum, cotton, potato, peanut, soybean, and sunflower. S-metolachlor, M-OXA and M-ESA are considered mobile in the environment [52]. The transformation products M-OXA and M-ESA are persistent compared to the parent compound, with *DT50*_*TP*_ values ranging between 12.2 and 27.2 days [53]. S-Metolachlor application rate (1.25g\ha) on maize and application frequency were obtained from the Austrian Federal Office for Food Safety (AGES) [54], which recommends the type of agrochemical for particular crops and the type of outbreak. Based on the AGES recommendations, 1.25 g/ha of S-Metolachlor was applied in the modelling framework at the beginning of each growing season in the first week of May. All the model parameterizations (Table 3) were run for one additional year (year 4) without agrochemical application as a buffer period to estimate the effect of agrochemical application after a cropping year.

**Table 3:** Input parameters of pesticide properties.

Compound	Variable	Value	Reference
S-Metolachlor	K_f_	3.63 (mL g⁻^1^)	[52]
	Solubility- water	480 (mg l⁻^1^)	
	DT_50-soil_	10.3	
Metolachlor Oxanilic Acid (M-OXA)	ff_PC-TP_	0.211	
	Koc	17 (mL g⁻^1^)	
	Solubility- water	360000 (mg l⁻^1^)	
	DT_50-soil_	12.2	
Metolachlor Ethyl Sulfonic Acid (M-ESA)	ff_PC-TP_	0.235	
	Koc	9 (mL g⁻^1^)	
	Solubility- water	212461 (mg l⁻^1^)	
	DT_50-soil_	27.2	

## 3 Results

### 3.1 Model calibration and validation

**Fig. 3:**
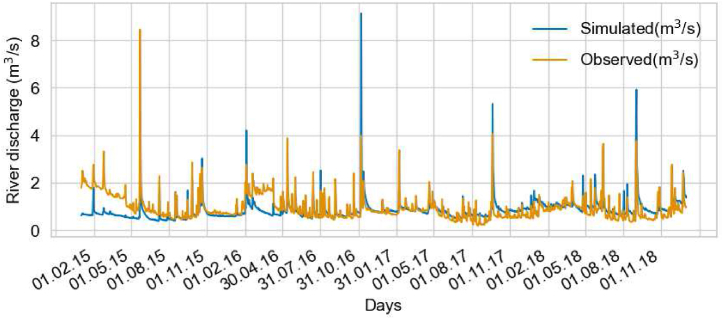
Comparison of the observed and simulated river discharge at the gauging station in Schützen am Gebirge, Austria (2015–2018).

The Zin-AgriTra model was calibrated using the Schützen am Gebirge daily river discharge data for 2015–2018, following an initial 2-year warm-up run. The model was manually calibrated with up to 60 runs. The selection of the best model run was made by a compromise between baseline flow and the height of the discharge peaks. The last three calibration runs from 01/2015-12/2018 were tested against discharge measurement data with the Kling-Gupta efficiency to choose the best model run with 0.52 efficiency [55,56] and are presented in Supplemental material-B.6 Model Calibration. The best model run is shown in Fig. 3 and reproduces the general hydrologic behaviour of the catchment and water balances (See Supplemental material-B.6 Model Calibration). The reduced discharge in the simulated discharge at the beginning of 2015 is due to lower initial soil moisture values used for the calibration to reduce the overestimation of peaks. The results indicate that the overall peaks in the observed discharge match the simulated discharge. However, the peaks in discharges of Feb 2016, Nov 2016, Oct 2017, and Sept 2018 are overestimated in simulated discharge (Fig. 3). Potentially, this is caused by the presence of a dam or weir that is not accounted for in the model.

### 3.2 Comparison of agrochemical fate and transport to different environmental compartments

**Fig. 4:**
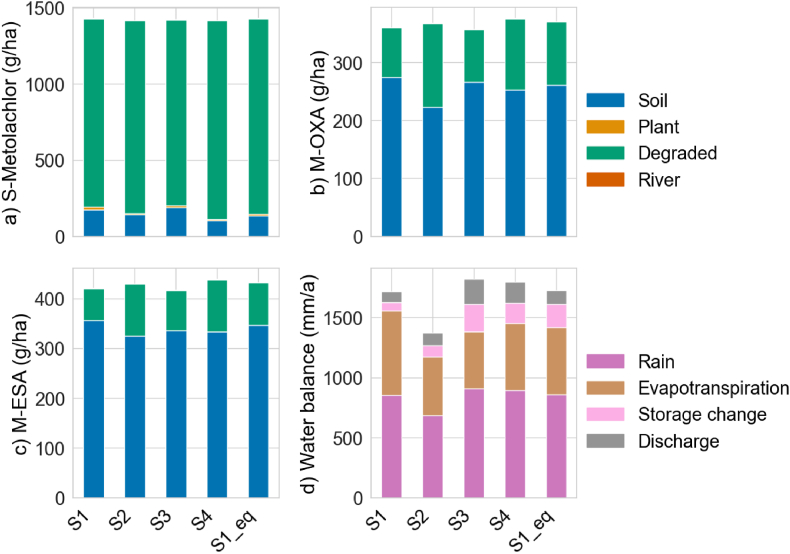
Shows the total balances of a) S-Metolachlor (g/ha), b) M-OXA (g/ha) and c) M-ESA (g/ha) at the end of three years remaining in the soil, up taken by plants, total degraded amount and exported via river channels. The total water balances of the catchment at the end of three years are presented in d) Water balance (mm/a). The scenarios represent four climate extremes: Reference as S1 and S1_eq, very dry as S2, very wet as S3 and highest temperature as S4.

The long-term influences of extreme climate events on agrochemical fate and transport are presented as the modelled balances and flows of S-Metolachlor, M-OXA and M-ESA in different environmental compartments. Fig. 4 shows the total balance of S-Metolachlor, M-OXA and M-ESA remaining in the soil and exported via river channels, the amount taken up by the plants and the part that is degraded. About 13–14 % of applied S-Metolachlor remains in the soil at the end of three years (Fig. 4a), and around 85 % is degraded to TPs. The degraded mass of S-Metolachlor in Fig. 4a is comparable to total M-OXA (Fig. 4b) and M-ESA (Fig. 4c) masses in soil, plant uptake, degradation, and export via river channels.

However, in comparison to S-Metolachlor, around 80 % of formed M-OXA and M-ESA remain in the soil, with less than 18 % of it degraded. MESA and MOXA are much more soluble and only weakly sorbed; they remain dominantly dissolved in the aqueous portion of the soils. Furthermore, only small masses of S-Metolachlor, M-OXA and M-ESA are transported to the river (0.3 %) or taken up by the plants (0.1 %). Up to 1.8 g/ha of S-Metolachlor is taken up by the plants (Fig. 4b) with even lower uptake for the TPs. The total S-Metolachlor transport to the river channel ranges between 1 and 3 g/ha and 0.5–1 g/ha for M-OXA and M-ESA, which is relatively low compared to the amount retained in the soil.

The overall trends in the four scenarios highly vary in different environmental compartments. The high-temperature scenarios have the lowest balance of S-Metolachlor remaining in the soil and a higher total degraded amount, which indicates that, with more high-temperature days, there is higher degradation (Fig. 4a). A similar effect is observed in the very dry scenario: with higher residence time, there is higher S-Metolachlor degradation and lower soil balances at the end of three years. On the contrary, the very wet scenario shows a high amount of S-Metolachlor in soil and exported via river channels but the lowest degraded amount due to higher agrochemical transport (Fig. 4a). Similar to S-Metolachlor, degradation of M-OXA and M-ESA is higher in high-temperature and very dry scenarios, with the latter having the highest temperatures after S4.

Although the majority of S-Metolachlor and TPs are retained in the soil or degraded, their dynamics highly vary in plant uptake and export to the river. The reference scenario S1 shows the most considerable uptake in plants, followed by the very wet scenario, due to higher evapotranspiration (Fig. 4d) and reduced leaching. During high-intensity rainfalls, the agrochemical is present mainly in the mixing (top) soil layers, making it readily available for plant uptake through evapotranspiration, especially in reference scenario S1, where the sampled hourly rainfall has high intensities compared to the disaggregated rainfall of the other scenarios. A comparable effect of high S-Metolachlor export via river is observed in very wet (S3) and reference scenario S1.

**Fig. 5:**
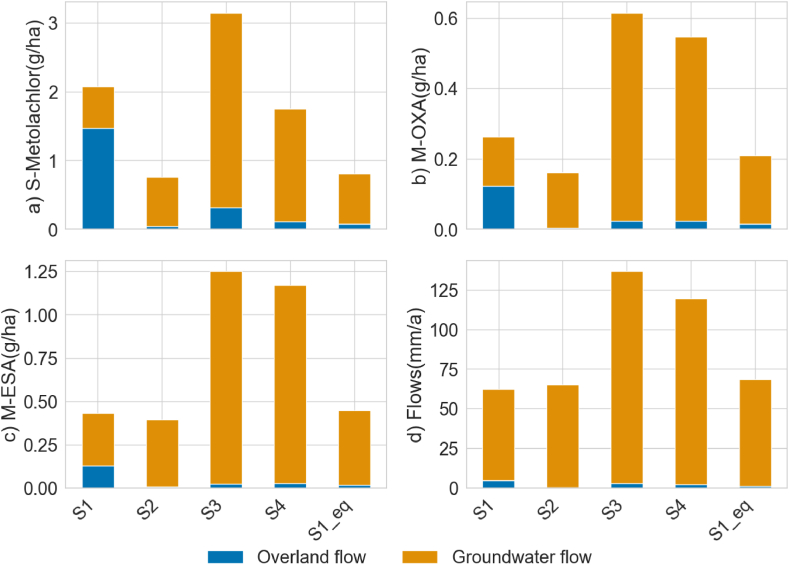
Shows the total export of a) S-Metolachlor (g/ha), b) M-OXA (g/ha) and c) M-ESA (g/ha) at the end of three years via overland flow and groundwater flow. The total water flow via overland and groundwater flow is presented as d) Flows (mm/a). The scenarios represent four climate extremes: Reference as S1 and S1_eq, very dry as S2, very wet as S3 and highest temperature as S4.

The main transport processes simulated after the S-Metolachlor application are presented in Fig. 5. The highest S-Metolachlor, M-OXA and M-ESA transport at the end of three years is observed in groundwater flow, followed by overland flow except for S1. The S-Metolachlor transport via groundwater flow is one of the main contributions to agrochemical concentrations in the river because of higher S-Metolachlor retention in soil (Fig. 4a). In contrast to overland flow, the total flows of M-OXA and M-ESA are higher in environmental compartments that have a relatively high residence time for degradation of S-Metolachlor, like in groundwater flow (Fig. 5b and (c)). Similarly, high concentrations of S-Metolachlor in overland flow, while M-OXA and M-ESA concentrations were higher in subsurface flow, were reported by Rose et al. [57]. The transport of S-Metolachlor, M-OXA and M-ESA via both overland and groundwater flow increases with higher-intensity rainfalls (See Supplemental material-A.1). Interestingly, on comparing daily overland and groundwater flows of S-Metolachlor, M-OXA and M-ESA in the four scenarios, it is seen that groundwater flow is higher in the buffer year (year-4) when agrochemical was not applied (See Supplemental material-A.1). The effects of agrochemical application on fate and transport processes can be observed even a year after application.

**Fig. 6:**
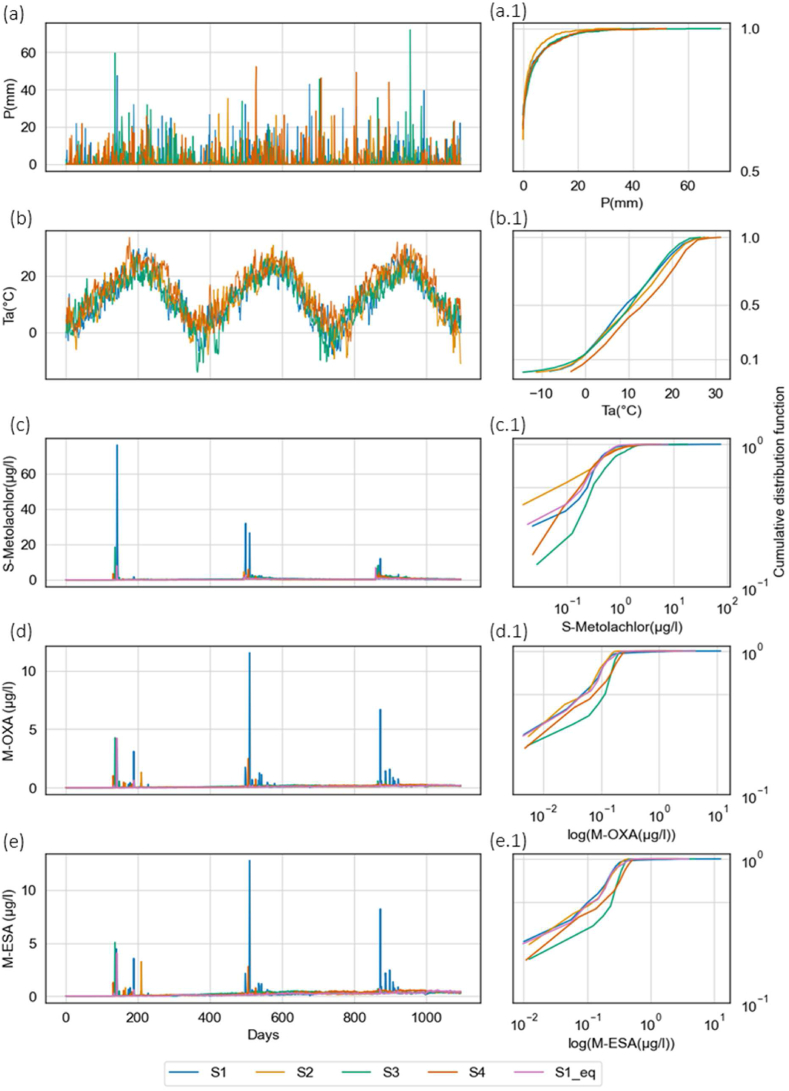
Presents the (a) daily precipitation in mm and (a.1) cumulative probability density of daily precipitation in mm, (b) daily average temperature in ^0^c and (b.1) cumulative probability density of daily average temperature in ^0^c, (c) daily S-Metolachlor concentrations in μg/l at the catchment outlet and (c.1) log cumulative probability density of daily S-Metolachlor concentrations in μg/l, (d) daily M-OXA concentrations in μg/l the catchment outlet and (d.1) log cumulative probability density of daily M-OXA concentrations in μg/l, and (e) log of daily M-ESA concentrations in μg/l at the catchment outlet and (e.1) log cumulative probability density of daily M-OXA concentrations in μg/l. The scenarios represent four climate extremes: Reference as S1 and S1_eq, very dry as S2, very wet as S3 and highest temperature as S4.

The total S-Metolachlor, M-OXA and M-ESA transport via overland and groundwater flow in different scenarios are identical to the total water flow through overland and groundwater flow (Fig. 5d). S-Metolachlor has higher export in overland flow for the reference scenario-S1 due to higher wash-off from the soil with intense hourly rainfall. In comparison to S1, S-Metolachlor export via overland flow in reference- S1_eq, very dry, wet, and high-temperature scenarios are relatively small due to distributed hourly rainfall throughout the day that reduces the peak wash-off. Though S1 and S1_eq have the same daily rainfall, S1 has transport flows like the very wet scenario because of intense hourly rainfall on S-Metolachlor transport (Fig. 6a). However, the S-Metolachlor export via overland flow in the very wet scenario is four times more compared to the very dry scenario, two times more compared to the high-temperature scenario and more than three times compared to reference scenario S1_eq. The higher overland and groundwater flows in very wet and high-temperature scenarios are associated with more rainfall days and high-intensity rainfall events in the scenario. However, the effect of high temperatures on S-Metolachlor, M-OXA and M-ESA degradation has not been substantial comparing the yearly balance of the scenarios. Comparing the daily degradation rates with days above and below 20 °C temperature, it is seen that with high temperatures, degradation rates of S-Metolachlor, M-OXA, and M-ESA are higher, specifically for the very dry, reference (S1_eq) and high-temperature scenario (See Supplemental material-A.2).

### 3.3 Short-term effects on agrochemical and TP fate and transport under climate scenarios

S-Metolachlor applied on the agricultural fields is transported to the catchment outlet along with the TPs based on hydrology and local environmental conditions. The short-term influences of extreme climate events on fate and transport are presented as the daily river concentrations of S-Metolachlor, M-OXA, and M-ESA (Fig. 6).

The daily precipitation in the very wet scenario shows the highest extreme precipitation events, followed by scenario S4, representing high temperature and reference scenario S1 (Fig. 6a), in which we have plotted daily values and cumulative probability density of the daily precipitation values. The average temperature difference between the scenarios is 0–10 °C (Fig. 6b & (b.1)). The three peaks in Fig. 6b represent the three growing seasons simulated. Differences between years and between scenarios in precipitation and temperature are given in Table 2.

The simulated S-Metolachlor concentrations at the outlet are shown in Fig. 6c. All scenarios simulated high peaks in S-Metolachlor concentration at the outlet straight after application to the agricultural fields at the beginning of each growing season. The number of days between application and peaks at the outlets varied between 4 and 15 days, depending on the scenario. The reference scenario (S1) shows the highest peak discharges, followed by the very wet scenario. For all scenarios and all years, the daily concentrations at the end of the year are extremely low compared to values during the growing season immediately after application. Hence, the S-Metolachlor concentrations look similar for all the scenarios (Fig. 6c). However, the daily S-Metolachlor concentrations varied in the four scenarios and are represented as cumulative probability density curves (Fig. 6(c.1)). The cumulative probability density of S-Metolachlor shows that the very wet scenario has higher daily average S- Metolachlor concentrations and higher peaks (Fig. 6(c.1)). While the very dry scenario has lower daily S-Metolachlor concentrations. Similarly, the reference scenarios S1 and S1_eq show lower daily S-Metolachlor concentrations due to a lower number of rainfall days. However, S1 shows higher peak S-Metolachlor concentrations due to high-intensity hourly rainfalls, contributing to high S-Metolachlor concentrations at the catchment outlet via overland flow (Fig. 5a). The higher S-Metolachlor concentrations observed in very wet and high-temperature scenarios are due to higher river discharges in the scenario. The S-Metolachlor concentration peaks reduced over time in all scenarios. However, they are still detected in 0.1–0.5 μg/l concentrations in the river discharge even after the cropping season. The seasonal patterns of S-Metolachlor concentrations after the application in agriculture and the formation of M-OXA and M-ESA for the remainder of the year were similarly observed in field studies [57]. Except for S1, where the S-Metolachlor peaks are higher due to overland flow, the simulated S-Metolachlor concentrations after the first rainfall event are comparable to field Metolachlor concentrations up to 10 μg/l, as reported by Boithias et al. [25]. However, the overestimation of S-Metolachlor concentration in S1 can be associated with the overestimation of river discharge peaks by the model.

The M-OXA (Fig. 6d) and M-ESA (Fig. 6e) concentrations at the catchment outlet show a small build-up, up to 0.5 μg/l, after three years of S-Metolachlor application. The concentration of M-OXA and M-ESA gradually increased throughout the growing season, with a build-up effect of up to 0.5 μg/l observed in the next year (See Supplemental material-A.3). M-OXA and M-ESA show the highest concentrations in very wet scenarios due to higher discharge similar to S-Metolachlor, followed by the higher temperature scenario. Compared to other simulation scenarios, the reference scenario-S1 shows higher peaks in M-OXA and M-ESA due to peak discharges associated with intense hourly rainfall generating higher overland flow. On the contrary, the scenarios with higher groundwater flow (S1_eq, S3, S4, Fig. 6b and (c)) show reduced peaks in concentration. In Fig. 6c, S-Metolachlor concentrations constantly decrease over time in the year due to DT50 ranging from 10.3 to 51 days. The M-OXA and M-ESA concentration peaks keep rising as there is constant input of TPs by the degradation of S-Metolachlor. Overall, the simulated pattern (rise and fall) in the simulated river concentrations of S-Metolachlor, M-OXA and M-ESA in the four scenarios corresponds to patterns in the river discharge.

## 4 Discussions

### 4.1 Understanding the dynamics of agrochemical fate and transport with climate change scenarios

Climate change effects were quantified with changes in rainfall and temperature in four scenarios in this study. The effects of extreme climate events on S-Metolachlor fate and transport are discussed as long-term and short-term impacts. The long-term impact on S-Metolachlor fate and transport in the four climate scenarios show huge differences in the balances and flows in different environmental compartments. Considering precipitation influences on long-term impact, we see that total S-Metolachlor transport to rivers is higher in the very wet and high-temperature scenario, which has higher frequency and intensity of rainfall. A similar effect is observed in reference scenario S1, which shows a larger overland flow due to intense hourly rainfall, which leads to a high exchange of S-Metolachlor and TPs in the thin upper soil layer, also known as the soil mixing layer due to interaction with surface runoff by sorption processes, thereby higher transport. On comparing reference scenarios S1 and S1_eq, which have the same daily rainfall but different hourly distributions, it is evident that rainfall intensity, even on an hourly scale, has a high impact on agrochemical transport to rivers, primarily via overland flow because of reduced deeper leaching due to soil saturation. This emphasises the need for climate model (RCM) results with a high temporal resolution for studies on the effects of climate change on water quality in order to correctly simulate the relevant hydrological transport processes. Additionally, with increasing amounts of rainfall, we see higher S-Metolachlor, M-OXA, and M-ESA transport in both overland flow and groundwater flow in all the scenarios (See Supplemental material-A.1). Since the simulation scenarios of very dry, very wet, and high temperatures use equally distributed hourly rainfall data, they underestimated the transport via overland flow. It is crucial to note that such an effect can become prominent during extreme climate events with high-intensity rainfalls. The general modelling trend of increased transport (via overland or groundwater flow) and, thereby, discharge of agrochemicals and TP with higher rainfalls is documented in the literature [14,25,58,59]. Therefore, we conclude that RCM simulated climate model data with high temporal resolution is necessary to understand the effects of climate change on pesticide and TPs river concentrations.

There are no huge differences in the short-term impacts on S-metolachlor concentrations in the different scenarios. However, we see that daily M-OXA and M-ESA transport peaks correlate with rainfall events. The frequency of such peaks is higher in the reference scenarios associated with higher overland flow caused by intense hourly rainfall (Fig. 6). It is essential to note that M-OXA and M-ESA are building up over the years, meaning that long-term application can lead to high concentrations even at the beginning of the next cropping year. Though, there are no major changes in the long-term dynamics of agrochemical fate and transport with temperature. The daily degradation rates increase with days with high temperatures (Supplemental material-A.2) due to higher microbial degradation [11,58,60].

Although the degradation fractions of S-Metolachlor are similar in the different scenarios, the highest degradation is observed in scenarios with a greater number of days above 20 °C, mainly in high temperature and very dry scenarios. In both very dry and high-temperature scenarios, we see lower S-Metolachlor and TP uptake by plants due to more high-temperature days. High temperatures lead to faster drying of the soil, hence lower crop transpiration and, thereby, reduced plant uptake. Additionally, it is expected that high temperatures promote plant growth, which leads to roots penetrating into deeper soil layers, preventing the uptake of agrochemicals present in the topsoil layers [61]. On the contrary, the plant uptake is higher in the reference and very wet scenario where the temperatures are comparatively lower, making the agrochemical readily available in topsoil layers for plant uptake. The presence of S-Metolachlor in the topsoil layer can also be concurred with higher S-Metolachlor export via overland flow in these scenarios. Furthermore, the increased plant uptake in the reference scenario is due to higher evapotranspiration in wetter conditions [62].

The effect of high temperatures on S-Metolachlor, M-OXA and M-ESA degradation has not been substantial, possibly due to several reasons. First, microbial degradation is calculated by multiplying the transformation rate with soil moisture, temperature, and depth [34], where other factors can weaken temperature effects. For example, when temperatures rise, soils might get drier due to higher evapotranspiration. Thus, lower soil moisture might compensate for the positive effect on microbial activity by higher temperatures. Second, the days with high temperatures are distributed throughout the year, which could balance out the high and low-temperature days on the yearly balance. Lastly, the agrochemical and TP transport is directly estimated with water movement from cell to cell when comparing rainfall effects. Meanwhile, the effect of temperature on agrochemicals or TPs depends on other environmental conditions.

The short-term and long-term effects of extreme climate events presented in the four scenarios are comparable with the literature. The simulated river concentrations of S-Metolachlor peaking immediately after application and dissipating over time are similar to Lutz et al. [63] and Rose et al. [57], irrespective of differences in the catchment. The first wash-off after application shows the highest S-Metolachlor concentrations at the river outlet, exceeding the permissible limits of 0.5 μg/l. However, we can argue that the peaks are unrealistically high since S-Metolachlor was applied at the same time on all agricultural fields in the catchment, which may not happen in reality. The effect of the first wash-off from the agricultural field having relatively higher S-Metolachlor concentrations in the year is documented similarly by Meite et al. [59]. The modelled pathways of S-Metolachlor fate and transport in the simulation scenarios were comparable to export pathways presented by Marie et al. [64] for the S-Metolachlor amount retained in the soil (13 %) and degraded (71 %).

### 4.2 Uncertainties in the modelling setup

The modelled agrochemical fate and transport uncertainties can originate at various stages. First, with the use of meteorological data in the reference scenario, due to data limitations, a single station was used to represent the whole catchment, which could undermine the spatial dynamics of climate parameters, especially for precipitation within the Wulka catchment area. For the climate scenarios up to 2100, climate data was obtained from downscaled RCM projections from RACMO22E, which has a higher spatial resolution for the catchment. Second, because of the complexity and spatial variability of the natural environmental conditions and related transport processes. It was impossible to achieve accurate model calibration that might influence predicted agrochemical fate and transport processes. Third, biases in the model parametrisations or setup compared to the reality, e.g., crop choice, either as grain maize or winter wheat, can alter fate and transport processes due to monthly changes in crop coefficients. Last, in the parameterisation of the S-Metolachlor, M-OXA and M-ESA properties. The DT50 values for the compounds vary widely between the lab and field conditions. The current parameterisation is based on the pesticide properties database. However, it is essential to note that DT50 measured ranges in the field can vary from 3.55 to 55.7 days [53] for S-Metolachlor, resulting from different environmental conditions such as soil moisture, soil temperature, organic carbon content, pH, and clay content. The data used in the model parameterisation is based on existing literature that represents S-Metolachlor and TP properties and can vary in the catchment. As the current research focuses on understanding the agrochemical fate and transport dynamics under extreme climate scenarios, S-Metolachlor is applied as an exemplary agrochemical, and actual concentrations under field conditions vary.

Although further model calibration with monitoring data could reduce uncertainties in the model parameterisation, Uncertainties can still originate from the inability of input parameters to describe field data. Beulke and Brown [65] emphasized this by highlighting the need for simulation models to adjust DT50 values to actual field conditions, as the laboratory DT50 values refer to specific temperature and moisture conditions. Degradation rates in the model equations can lead to much longer half-lives under varying temperature and moisture conditions during the simulations. The same is true for Koc values, where the simplified assumption that organic carbon is the only adsorbent often leads to wide ranges of values in the experimental literature [66]. Calibration of pesticide and TP fate models can be even more exacerbated by the interaction of parameters during calibration and by uncertainty propagation from the parent compound to the TP [29].

### 4.3 Qualitative assessment of agrochemical use, fate, and transport under climate change in the study context and its limitations

Understanding and modelling the fate and transport of agrochemicals and TPs in the environment under climate change is crucial for sustainable agricultural management. The current modelling framework presents the beginning steps for understanding agrochemical dynamics under extreme climate events. Although the modelling framework results applied in this study focus on a single herbicide, the primary outcomes can be inferred to other agrochemical categories. For example, where herbicides are used as seed treatments or after sowing, other agrochemicals such as insecticides and fungicides are applied later in the cropping season. Depending on the application timing in the cropping season, fate and transport processes vary with the crop growth stage (and related crop coefficients), which reduces agrochemical transport in overland flow and soil retention. The agrochemical application in the mid-season may result in either increased agrochemical and TP degradation or increased export dependent on high temperatures or shorter extreme rainfalls. Depending on the agrochemical substance properties, such as persistence and mobility, the agrochemical fate and transport processes can also substantially vary.

Additionally, several factors can further drive agrochemical use, which is not considered in our modelling approach. Due to climate change, the occurrence of pests, diseases, and weeds has significantly altered crop production in many regions [67,68], challenging crop protection and related pesticide application. Herbicide resistance is becoming a growing problem [[69], [70], [71], [72]], which is often overcome by an increase in the frequency of agrochemical usage [73]. Increasing insect pest outbreaks have been a massive problem in the past decades characterized by global warming, such as in Europe [74], especially due to an increase in overwinter pest survival rate, which can allow additional pest generations in a growing season [61,[75], [76], [77], [78]]. Rising pest pressure by already established or even new pests moving into cropping regions [79] increases agrochemical application rate and frequency and changes its spatial application areas [58,80] and, thereby, environmental exposure. Increasing application rates and frequency leads to higher agrochemical and TP concentrations in water bodies. In addition to direct impacts, there may be significant indirect impacts of climate change on agrochemical use, fate, and transport due to changes in cropland use [81], choice of crops [17,82,83], agricultural practices and land management [21,75,81,84], and soil functions [85].

## 5 Conclusion

We presented an integrated modelling framework for assessing the impacts of extreme climate events on agrochemical fate and transport by coupling the Zin-AgriTra model with climate records and scenario (RACMO22E RCP 8.5) periods. Based on the results, we can infer that the integrated modelling framework helps to assess the short-term and long-term effects of extreme climate events through four applied simulation scenarios. The results of the applied modelling framework for a selected case study catchment present new insights for understanding agrochemical dynamics under different climatic characteristics of selected time periods of a climate scenario run for the 21st century. In general, the overall effect of different extreme climate conditions is likely very variable due to the uncertainties associated with hourly climate data and complex to predict due to the complexities in the model parameterisation of the catchment and, most importantly, agrochemical properties.

The short-term (daily) effects of extreme climate events on agrochemical fate and transport are comparable with the intensity and frequency of rainfall and high-temperature days. High-intensity hourly rainfall can cause higher peaks in agrochemical river concentrations due to overland flow. Meanwhile, the agrochemical and TP concentrations are higher in very wet and higher temperature scenarios due to high rainfall amounts and, thereby, river discharge. With increasing instances of extreme climate events, such an effect can become prominent. Hence, high temporal resolution simulated RCM climate data is vital to understanding the effects of climate change on pesticide and TPs river concentrations.

The long-term impacts indicate that agrochemicals like S-metolachlor end up in different environmental compartments once applied. Depending on climatic conditions, they are either retained in the soil (adsorbed or dissolved phase), taken up by plants or transported to ground or surface water. Plant uptake is higher in high-intensity rainfall scenarios due to increased evapotranspiration [62], such as in reference-S1 and very wet scenarios. The agrochemical and TP transport via overland and groundwater flow is more in very wet and high-temperature scenarios with higher total rainfall. In very dry and high-temperature scenarios, less agrochemicals are retained in the soil as degradation is higher due to more high-temperature days. Even though S-Metolachlor degrades over time, a small share of it is retained at the end of the year, which results in its continuous transport of TPs even in the subsequent years, specifically the buffer year when no agrochemical is applied. Though degradation of S-Metolachlor is better for the environment, the formation of TPs that are retained in the soil may negatively impact the environment.

## CRediT authorship contribution statement

**Poornima Nagesh:** Writing – review & editing, Writing – original draft, Visualization, Methodology, Formal analysis, Conceptualization. **Matthias Gassmann:** Writing – review & editing, Validation, Software. **Josef Eitzinger:** Writing – review & editing, Supervision, Methodology. **Hugo J. de Boer:** Writing – review & editing, Supervision. **Oreane Y. Edelenbosch:** Writing – review & editing, Supervision. **Detlef P. van Vuuren:** Writing – review & editing, Software. **Stefan C. Dekker:** Writing – review & editing, Supervision, Methodology, Conceptualization.

## Declaration of competing interest

The authors declare that they have no known competing financial interests or personal relationships that could have appeared to influence the work reported in this paper.
