# Supplementary material for: Modelling the impacts of climate change on agrochemical fate and transport by water on a catchment scale
Source: Heliyon. 2024 Aug 3;10(15):e35669. doi: 10.1016/j.heliyon.2024.e35669 (PMC11336872; doi:10.1016/j.heliyon.2024.e35669)

Modelling the impacts of climate change on agrochemical fate and transport by water on a catchment scale.

Supplementary Material- A

The summary of long-term and short-term effects of extreme climate scenarios of S-Metolachlor, M-OXA and M-ESA fate and transport are presented below. First, the balances and flows in different environmental compartments with the intensity of rainfalls are presented in the four scenarios (A.1). Second, the degradation of S-Metolachlor, M-OXA and M-ESA in comparison to daily average temperatures in different scenarios are shown (A.2). Third, the short-term adsorbed and dissolved river concentrations of S-Metolachlor, M-OXA and M-ESA at the catchment outlet are presented (A.3). Last, the river concentrations of S-Metolachlor, M-OXA and M-ESA at the catchment outlet for different scenarios showing a small build-up effect of transformation products over the years are presented (A.4).

1. Comparison of S-Metolachlor, M-OXA and M-ESA balances and flows with the intensity of rainfall in the four scenarios.
   1. Current scenario (2015-2018)


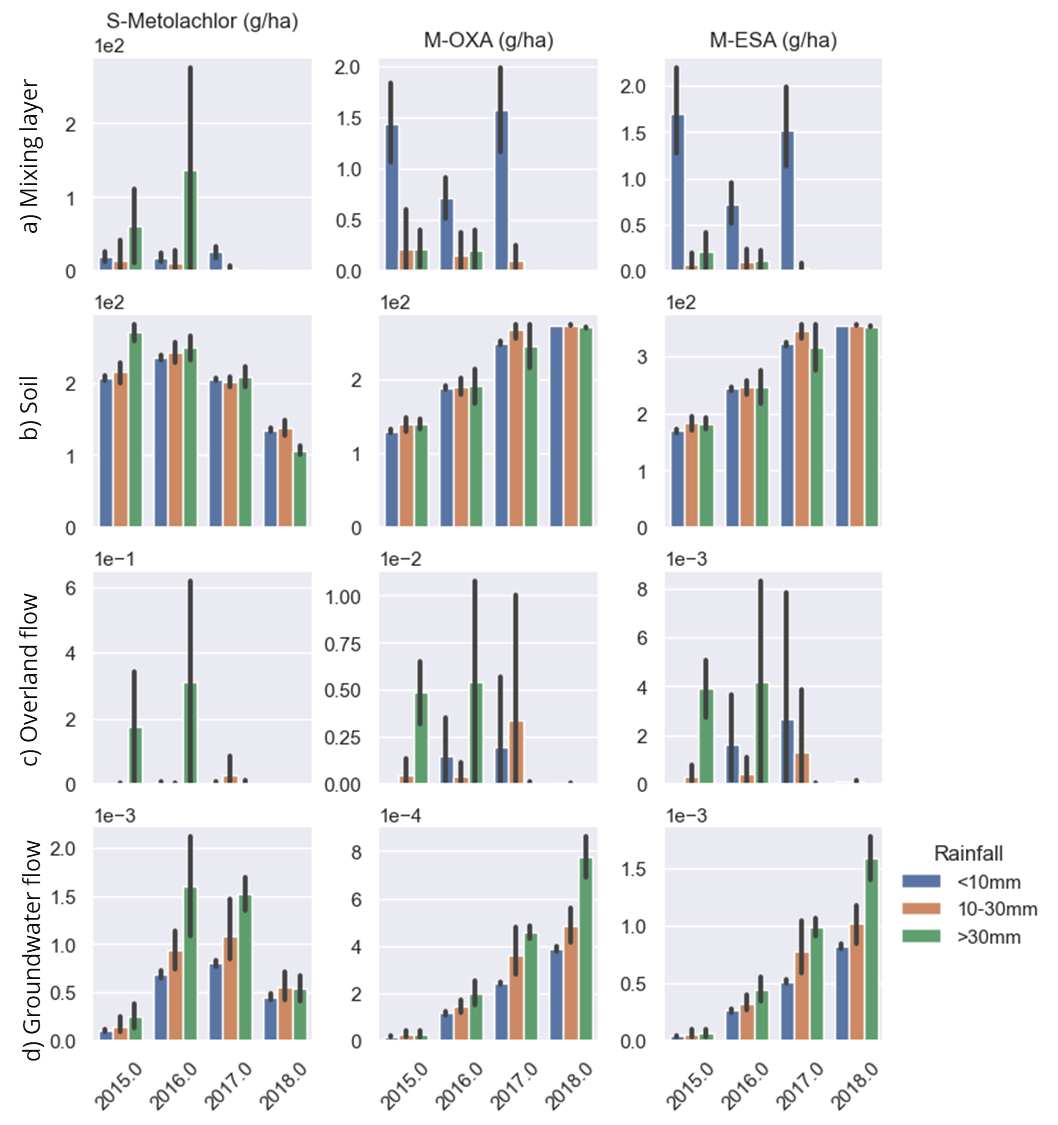


- 1. Very dry scenario (2038-2041)


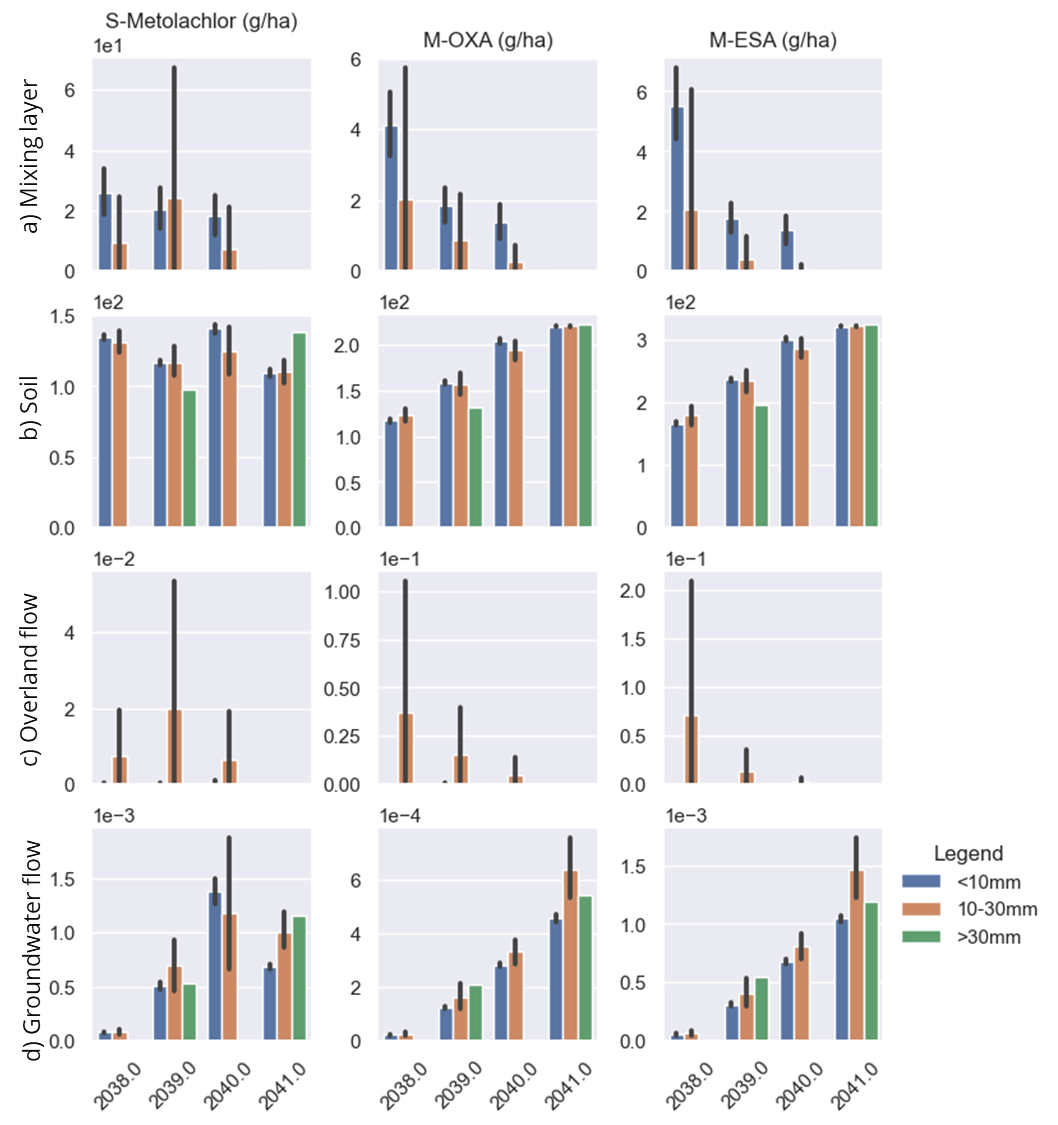


- 1. Very wet scenario (2054-2057)


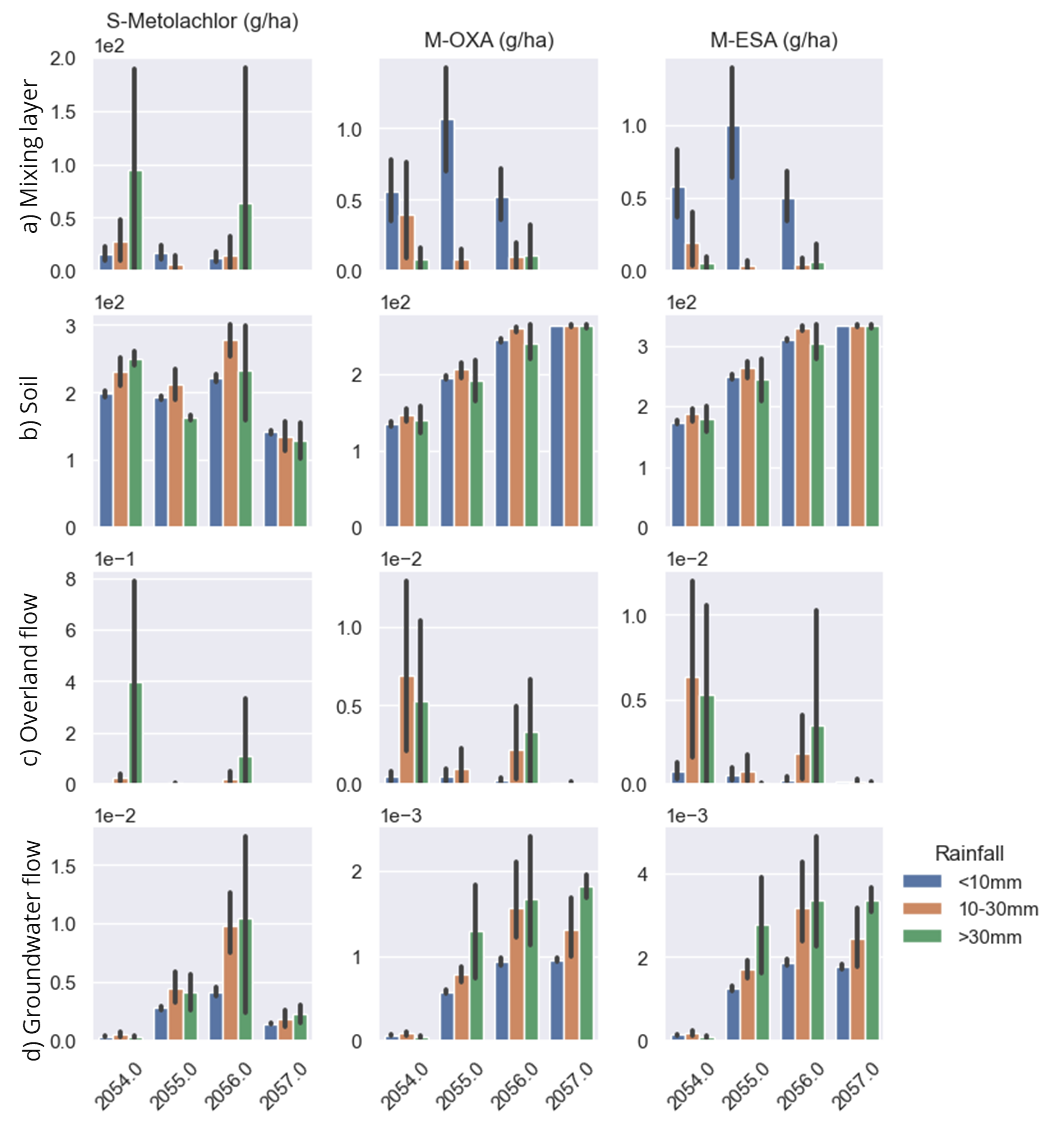


- 1. High-temperature scenario (2096-2099)


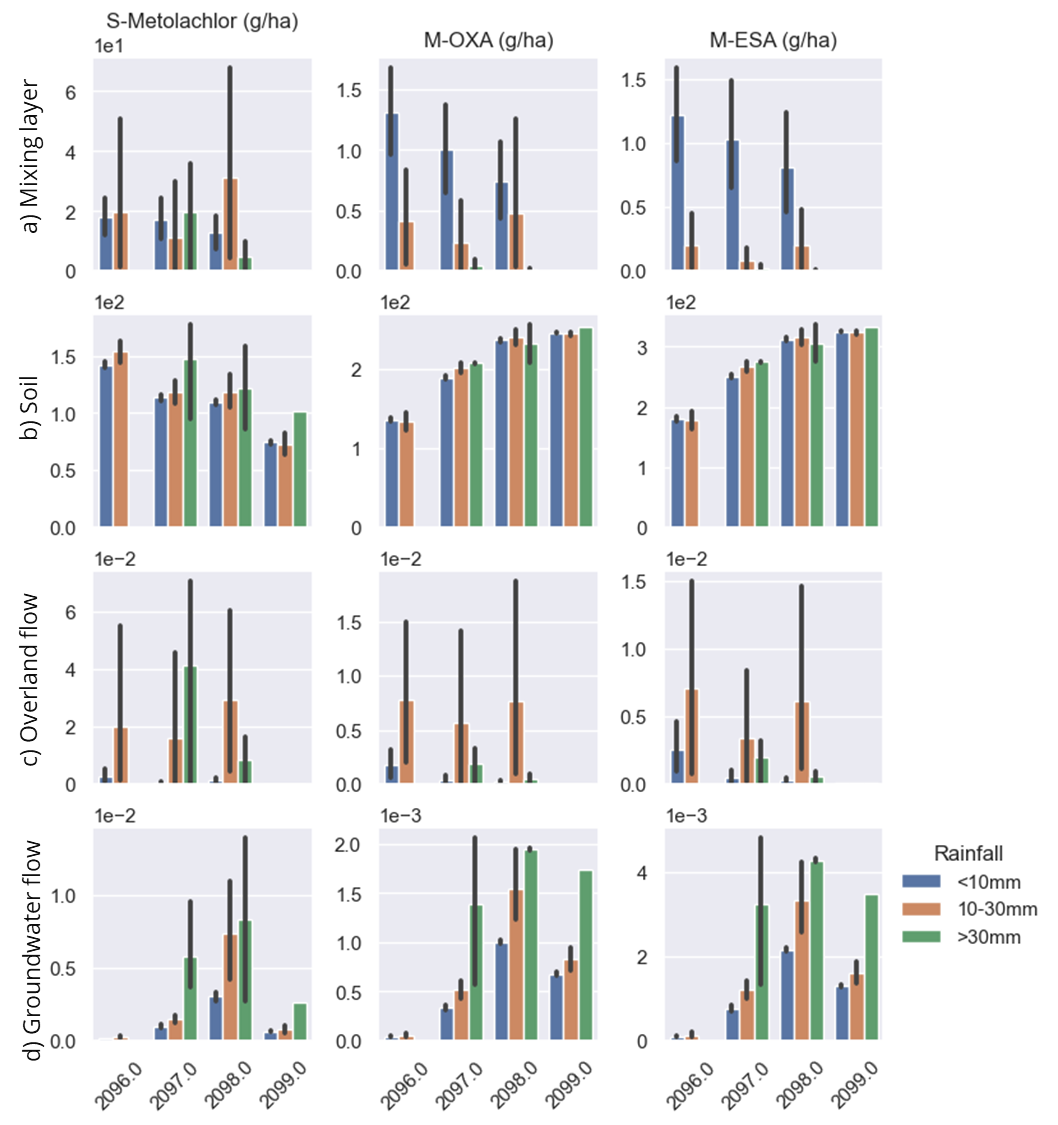


1. Comparison of S-Metolachlor, M-OXA and M-ESA degradation with increasing temperature in the four scenarios.


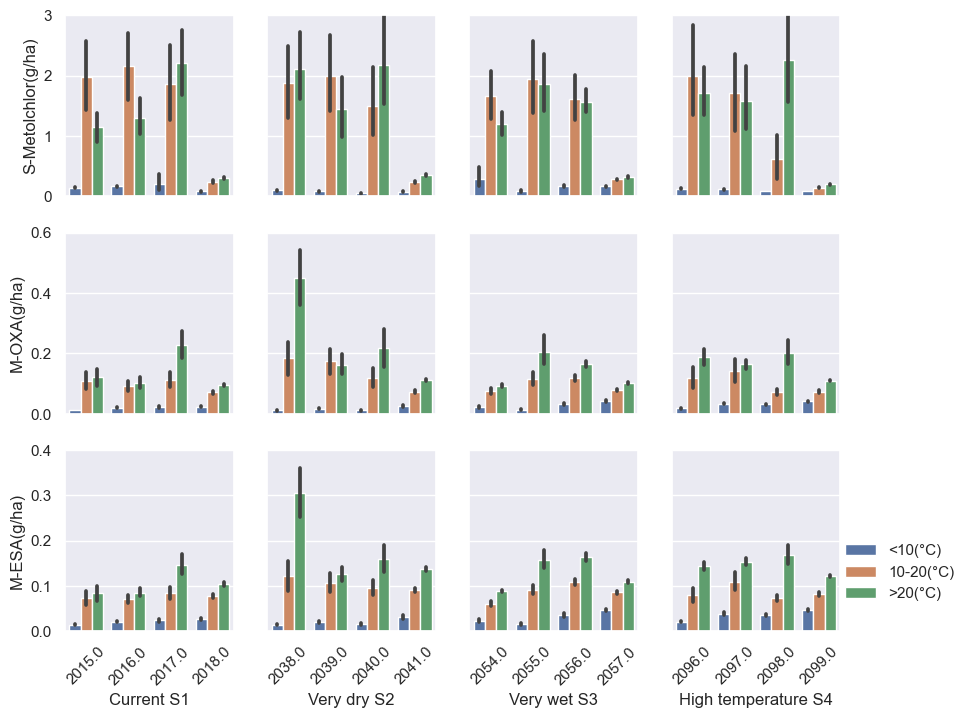


1. Comparison of dissolved and sorbed river discharge concentrations of S-Metolachlor, M-OXA and M-ESA with the intensity of rainfall in the four scenarios.


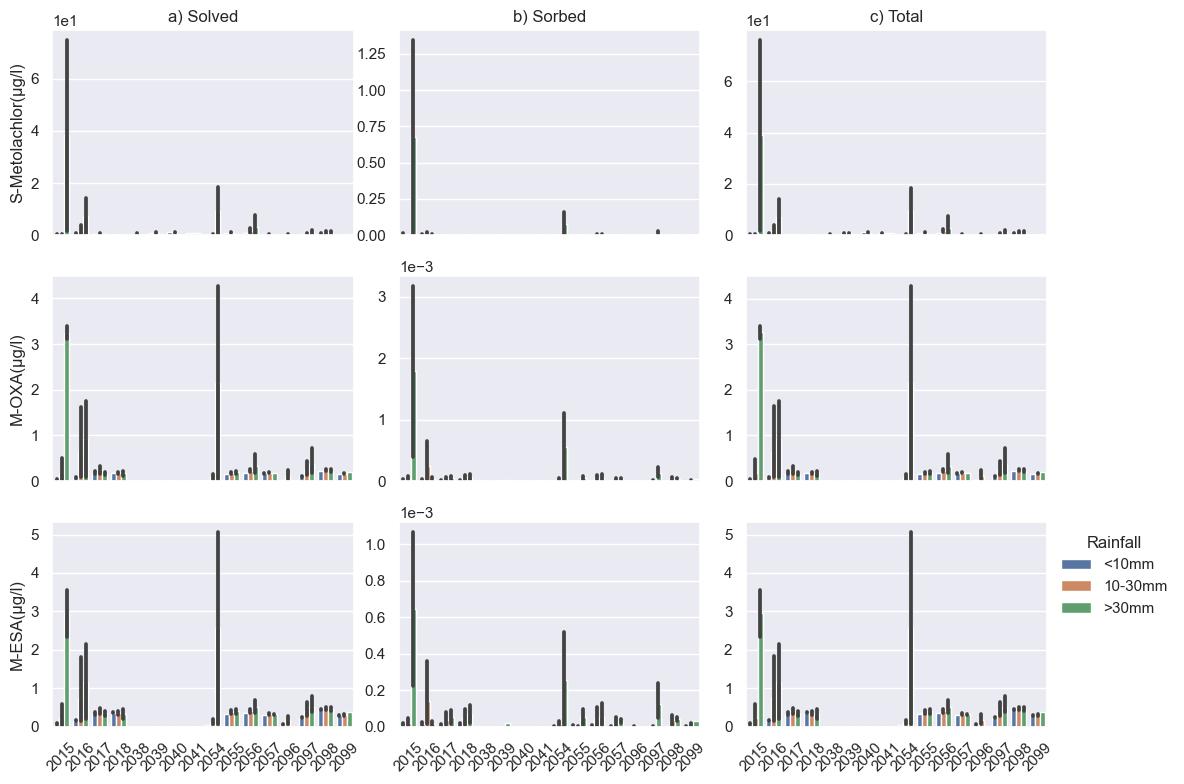


1. The river concentrations of S-Metolachlor, M-OXA and M-ESA on a log scale at the catchment outlet for different scenarios show a small build-up in the transformation products.


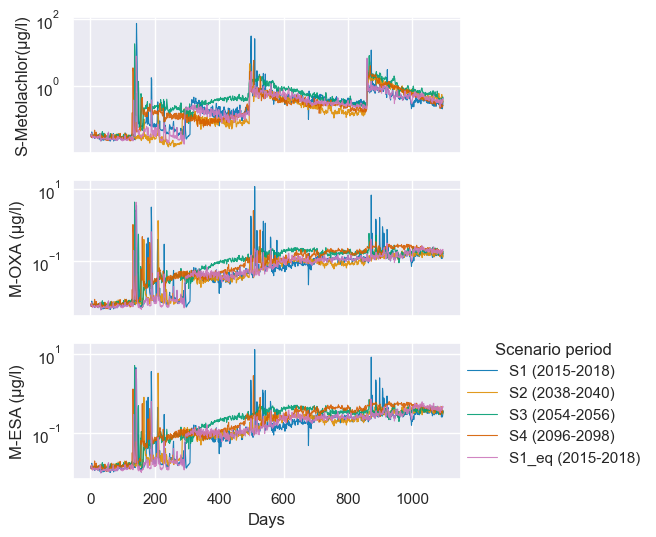

Supplement: Multimedia component 1 [file mmc1.docx]
